# Histological Study of the Toxic Effects of Solder Fumes on Spermatogenesis in Rats

**Published:** 2011-04-21

**Authors:** Mohammad Reza Arab, Mohammad Hossein Heidari, Rezvaneh Mashhadi, Ramazan Mirzaei, Mehdi Jahantigh

**Affiliations:** 1. Anatomy Department, Cell and Molecular Research Center, Faculty of Medicine, Zahedan University of Medical Sciences, Zahedan, Iran; 2. Basic Sciences Department, Faculty of Paramedicine, Shahid Beheshti University of Medical Sciences, Tehran, Iran; 3. Zahedan University of Medical Sciences, Zahedan, Iran; 4. Faculty of Health, Zahedan University of Medical Sciences, Zahedan, Iran; 5. Pathology Department, Faculty of Medicine, Zahedan University of Medical Sciences, Zahedan, Iran

**Keywords:** Spermatogenesis Index, Testis, Rat, Solder Fumes

## Abstract

**Objective:**

Toxic fumes generated during the soldering process contain various contaminants released at sufficient rates to cause both short- and long-term health problems. Studies have shown that these fumes change the quality and quantity of semen fluid in exposed workers. The aim of the present study was to determine the potentially toxic effects of solder fumes on spermatogenesis in seminiferous tubules of rats as an experimental model, with conditioned media in an exposed chamber.

**Materials and Methods:**

A total number of 48 male Sprague Dawley adult rats were randomly divided into experimental (n=30) and control (n=18) groups. Based on exposure time, each group was further subdivided into two, four and six subgroups. Rats in the experimental groups were exposed to solder fumes in an exposure chamber for one hour/ day. The concentrations of fumes [formaldehyde, stanum (Sn) and lead (Pb)] were measured by a standard method via atomic absorption and spectrophotometry. According to a timetable, under deep anesthesia, the rats of both experimental and control subgroups were killed. After fixation of testes, specimens were weighed and routinely processed. Paraffin sections were stained by hematoxylin and eosin. Spermiogenesis index was calculated and data analyzed by Mann Whitney NPAR test.

**Results:**

Analysis of air samples in the exposure chamber showed the following fume concentrations: 0.193 mg/m^3^ for formaldehyde, 0.35 mg/m^3^ for Sn and 3 mg/m^3^ for Pb. Although there was no significant difference in testes weight between control and experimental subgroups, there was only a significant difference in spermiogenesis index between the six week experimental and control subgroups (p<0.02).

**Conclusion:**

The results of this study showed that solder fumes can change the spermiogenesis index in experimental groups in a time dependent manner.

## Introduction

Fumes generated during metal welding have toxic effects on the human body. The types and quantity of such effects depends on the density and duration time of exposure to the fumes. Moreover, the types of fumes generated during welding or soldering are dependent on the electrodes or wires used. These fumes cover a wide spectrum, from formaldehyde to metal fumes such as lead (Pb) and stanum (Sn). Exposure to such pollutants, particularly insufficient ventilation of workplaces, increases the concentrations of fumes in breathing air and hence can increase health risk factors for workers (1). Epidemiological studies have indicated that these gases and fumes can seriously endanger the health of workers (2). Although there have been numerous studies conducted on determining the toxic effects of welding fumes on germinal epithelium in seminiferous tubules, no general consent has been achieved on the probable mehcanism of the fumes on seminiferous tubules as well as the quantity and quality of the produced spermatozoa (3, 4). An increase in infertility rate from 8% to 15% during the last decades has brought about serious questions on the impact of such toxic effects as one of the major reasons for infertility; therefore, a need exists for serious studies on this issue (5). Studies have shown both a decrease in the number of spermatozoids and their speed of movement in workers contaminated with Pb (5). When existing workplace pollutants are inhaled, respiratory air enters the blood stream after being taken up by the digestive and respiratory systems; hence, these pollutants systemically cause tissue damage (6, 7). It has been shown that metal fumes and gases generated during welding due to an increase in their temperature are released into the air; after reacting with oxygen, they produce metal oxides responsible for tissue toxicity (8). Fumes generated during the welding process can produce eye, skin and upper respiratory tract inflammations. Studies have shown that these pollutants may reside in the nasal epithelium (9). Decreased spirometry indexes in welders or solders in electronics and telecommunications industries have occurred following inhalation of toxic fumes (10).

The germinal epithelium is a pseudostratified epithelium responsible for production of spermatozoids in seminiferous tubules. These tubules are composed of two types of cell populations: a spermatogenic cell line and sertoli cells. Consecutive cell divisions in the germinal epithelium along with the complex cell differentiation process of spermiogenesis are underlying mechanisms for the production and release of spermatozoa from sertoli cells. Therefore, the epithelium is a suitable target for toxic agents, which are called gonadotoxins (11, 12). Workplace exposure to these gonadotoxins is a major concern of increasing importance in medicine. The Institute of Occupational Safety and Health has introduced infertility due to gonadotoxins as a major research subject. Gonadotoxins include a wide spectrum of metal fumes, insecticides and other solvents that seem to seriously damage the spermatogenesis in workers in the reproductive stage (11). Studies have shown that Pb toxicity influences the hypothalamohypophysial axis and is one of the main toxicity pathways in the testes; hence it is a high risk for welders or solders (13).

The present study determined the potentially toxic effects of inhalation of solder fumes on spermatogenesis in seminiferous tubules of rats as an experimental model, with conditioned media in an exposed chamber. We evaluated the spermiogenesis index as one of the testis functional indexes of spermatogenesis in rats.

## Materials and Methods

A total number of 48 Sprague Dawley adult male rats purchased from Pasture Institute (Tehran, Iran) were divided into experimental (n=30) and control (n=18) groups. After adaptation to standard laboratory conditions (12 hours light/12 hours dark; humidity 45% - 50%; 22 ± 2℃; free access to food and drinking water), experimental and control groups were equally subdivided into two, four and six week subgroups. Experimental groups were exposed to colophony solder flux fumes generated manually for one hour/day (13:00 - 14:00) and directed into a plexy glass exposure chamber. The exposure chamber had an internal volume of 0.83m^3^ connected to a 200 cm long hood inlet and outlet, which was ventilated 5 - 6 times per hour. The feeding rate of the manual solder wire was 5 m/minute. The chamber temperature was 22 ± 2℃. Zahedan University of Medical Sciences Ethics Committee approved the experimental design.

Air samples from the exposure chamber were collected daily using the SKC personal pump (SKC 224-EE, UK) and analyzed for fume concentrations of formaldehyde, Sn and Pb. The concentrations of solder fumes were 0.193 mg/ m^3^, 0.35 mg/m^3^ and 3 mg/m^3^ and for formaldehyde, Sn and Pb, respectively. All measurements were obtained in accordance with methods described by the National Institute of Occupational Safety and Health (ASTM, D4185-90, NIOSH 3500 and OSHA 206) using a visible absorption spectrophotometer (Spectronic 20D, Milton Ray, Belgium) and atomic absorption spectrophotometer (ATI/Unicam, 929, USA) (14-16). The soldering wire (alloy 63/67, 0.8 mm diameter, Jarfe Company, Iran) was commercially available. According to the study timetable, rats in both experimental and control subgroups were kept under deep anesthesia and the left testes removed and weighted.

After fixation in 10% formalin saline, tissue specimens were processed routinely and prepared paraffin sections (5-7µm thickness) stained by hematoxylin and eosin. The spermiogenesis index was calculated blindly in all prepared sections as the percentage of seminiferous tubules with spermatozoid in at least 200 tubules. Histological sections were examined for any histological changes in seminiferous tubules and interstitial tissues as qualitative variables. We analyzed obtained data with the Statistical Package for Social Sciences (SPSS, version 13), NPAR test of Kruskall Wallis and Mann Whitney U test. P values less than 0.05 were significant.

## Results

Although there was a slight increase in the weight of testes between the experimental and control subgroups, statistical analysis of obtained data for the testes weights showed no statistically significant differences between all subgroups. Analysis of data for spermiogenesis index showed only a statistically significant difference between the six week experimental and control subgroups (p<0.02, Table 1).

**Fig 1:**
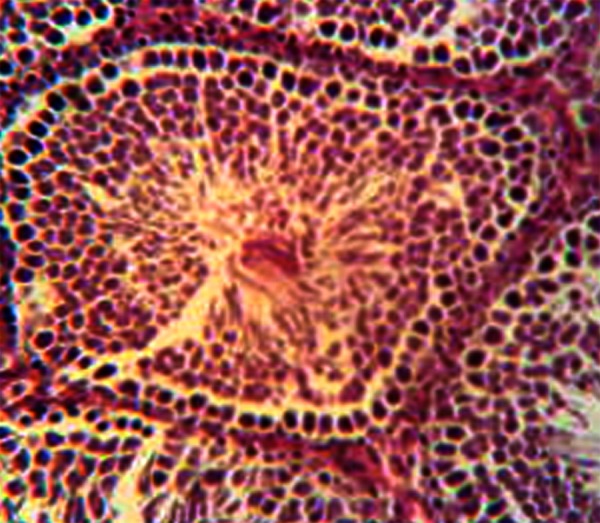
Normal architecture of germinal epithelium in two week control subgroup. H&E, magnification ×200.

**Fig 2:**
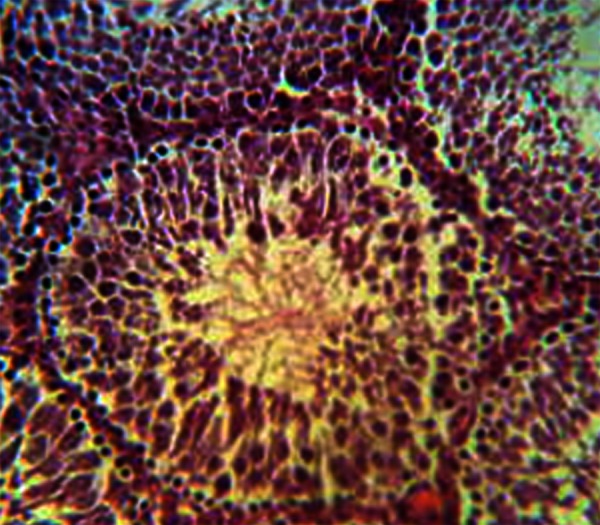
Slightly disorganized architecture of germinal epithelium and mild dilatation of blood vessels in two week experimental subgroup. H&E, magnification ×200.

**Fig 3:**
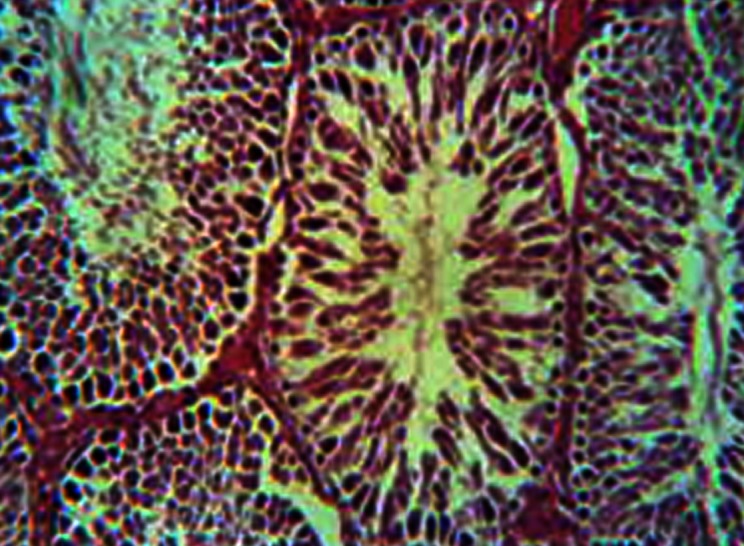
Intercellular spaces due to loss of intercellular junction and changes in staining properties of the germinal epithelium in the four week experimental subgroup. H&E, magnification ×200.

**Fig 4:**
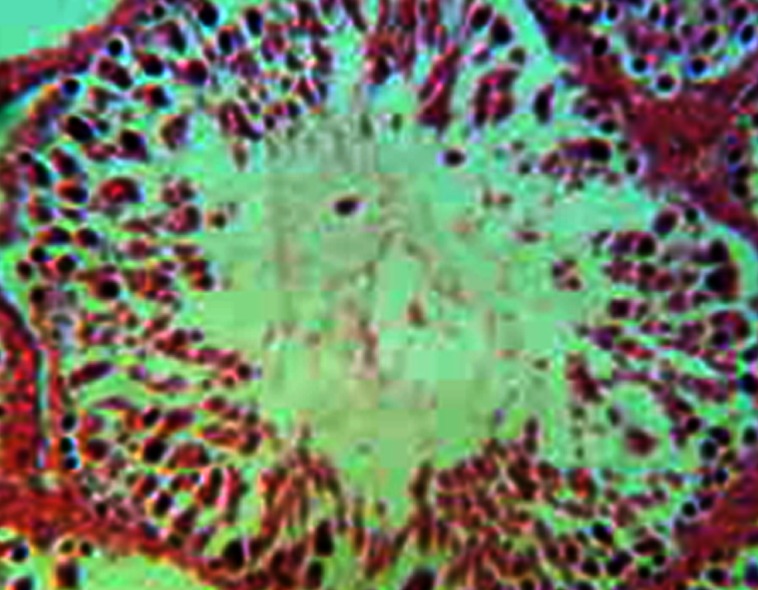
Severe disorganized architecture of germinal epithelium in six week experimental subgroup. H&E, magnification ×200.

Histological examinations of microscopic slides showed numerous structural changes in experimental subgroups in comparison to the control subgroups. These structural changes contained a wide spectrum of alterations that included dilatation of blood vessels, disorganized architecture of germinal epithelium, loss of intercellular junction between sertoli cells and spermatogenic cell line, decrease in height of germinal epithelium and some changes in staining properties of sertoli cells and the spermatogenic cell line (Figs 1-4).

Results showed the extent of these structural changes to be dependent on the exposure time. Analysis of air samples in the exposure chamber showed concentrations of fumes to be 0.193 mg/m^3^ for formaldehyde, 0.35 mg/m^3^ for Sn and 3 mg/m^3^ for Pb, which remained fixed during the experiment.

**Table 1:** Comparison of the obtained data between experimental and control subgroups after exposure to solder fumes.


Groups	Two week subgroup		Four week subgroup		Six week subgroup	
Variables	Control	Esperimental	Control	Experimental	Control	Experimental

Spermiogenesis index	84 ± 2	89 ± 1	92 ± 2	93 ± 3	97 ± 1*	75 ± 1
Testis weight	2.02 ± 0.36	2.23 ± 0.36	2.74 ± 0.36	2.07 ± 0.32	1.8 ± 0.16	1.99 ± 0.23


Data reported as mean ± SD, *p<0.02.

## Discussion

The results of the present study indicated that despite a non-significant difference between all studied groups in testes weights, there was only a significant difference in the spermiogenesis index for the six week experimental and its control group following the inhalation exposure of soldering fumes. A comparison of the qualitative parameters in the experimental and control groups showed that the fumes brought about numerous structural changes in the germinal epithelium of seminiferous tubules whose severity may be a function of the inhalational period. Deformity of the structural changes in the germinal epithelium and in the vascular connective tissue between the tubules and vasodilatation was more prominent in the experimental groups than the controls. The results showed a complete change in the connections of the lateral surfaces of the spermatogenic cell line with sertoli cells. Our results have shown that solder fumes can make numerous structural changes in the testis, though the mechanism of such changes is still unclear.

One of the limitations for determining the tissue effects of atmospheric fumes in workplaces is the changing condition of ventilation quality. The same issues along with other background and confounder variables in workers are the presence of other polluting agents (chemical solvents or smoking habits), which may increase the complexity of the problem (17). In the present study, we removed these variables by controlling the type and density of the fume in the exposure chamber. Analysis of the sampled gases in the exposure chamber showed that the amount of toxic fumes exceeded the standard level of 2 mg/m^3^ for Sn and 0.05 mg/m^3^ for Pb in the workplace. Fume concentrations remained fixed throughout the experiment.

Studies have shown that the toxic effects of organic tin compounds to be more than its nonorganic compounds. The inhibition of hydrolysis of adenosine triphosphate and interruption in oxidative phosphorylation in mitochondria were the main mechanisms for tin tissue toxicity (17). Possibly in our study, the non-significant differences between the control and experimental groups at two and four weeks resulted from low exposure time and small sample sizes.

Our previous studies have shown that pollutants generated during iron electric welding, are able to cause numerous structural and biochemical changes in the germinal epithelium of seminiferous tubules. Iron electric welding produces a wide spectrum of metal fumes including iron, copper, magnesium and chrome along with gases such as carbon monoxide, carbon dioxide, nitrogen oxides and ozone. Moreover, after inhalation exposures to those fumes, the pattern of reaction of the spermatogenic cell line and sertoli cells changed to lectins (18). Structural changes of germinal epithelium comprise changing cell surface glycoconjugates, an increase of the connective tissues in the periphery of seminiferous tubules, or changes in cell adhesion among the spermatogenic cell line and sertoli cells. These are among the clear tissue changes resulting from cytotoxic effects of pollutants. Although the exact mechanisms of their effects are unclear, it seems the mode of the action of each of these fumes may be different.

Stoy et al. have shown that increased temperature in workplaces may enhance similar tissue changes, which may lead to changes in the quality and quantity of the produced seminal fluid (19). Since the temperature in the gas room was constantly controlled in the present study, thus the observed tissue changes could not be attributed to temperature increase. Jung et al. have shown that interruptions in spermatozoid production in welders may not be due to temperature changes in the scrotum (20). It has been suggested that structural changes in the testes and related changes of the spermogram in workers may probably be due to systemic or local effects of fumes on the testes. Studies have indicated that sub-acute lead intoxication is capable of bringing about numerous structural changes in testes, which all may be dose dependent due to the additive actions of fumes. Structural changes of the seminiferous tubules, as well as a reduction in the number of sertoli and spermatozoid cells are among the most well known changes. The mechanism of such changes in testes may be related to the apoptosis pathway (21).

Although in the present study there were no significant differences in the testes’ weights in both the experimental and control groups, a study by Wang et al. has shown a significant reduction. This difference may be due to the mode of lead introduction in the lab animals (22). On the other hand, the interruptions in spermogram parameters following the effect of these fumes may be a reflection of changes in the spermatogenesis indexes as a basic mechanism for such changes.

It has been shown that formaldehyde, as one of the welding pollutants, can reduce the activity of superoxide dismutase enzymes, glutathione peroxidase and glutathione. Formaldehyde also increases the activities of malondialdehyde. Formaldehyde causes numerous structural changes, such as atrophy and structural deformities of the seminiferous tubules (23). Vitamin E can have a protective effect against the cytotoxic effects of these pollutants (23).

Heavy metal toxicity varies from species to species (24). Studies have shown that heavy metals such as Pb cause an irreversible toxic insult to the male reproductive system. Pb toxicity in the male reproductive system is manifested by the deposition of Pb in testis, epididiymis, vas deferens and semen ejaculate (24). Fumes produced during the soldering or welding process can change the structure of the germinal epithelium in the seminiferous tubule, and it seems these changes are the basic mechanism for changes in the quality and quantity of seminal fluid in exposed workers.

## Conclusion

Solder fumes have numerous effects on germinal epithelium in the testes. The extent of these changes is dependent on exposure time.
